# Theoretical and Experimental Study on AlGaN/GaN Schottky Barrier Diode on Si Substrate with Double-Heterojunction

**DOI:** 10.1186/s11671-020-03376-z

**Published:** 2020-07-16

**Authors:** Tao Sun, Xiaorong Luo, Jie Wei, Chao Yang, Bo Zhang

**Affiliations:** State Key Laboratory of Electronic Thin Films and Integrated Devices, University of Electronic Science and Technology of China, Chengdu, 610054 China

**Keywords:** GaN, Schottky barrier diode (SBD), Breakdown voltage, Silicon substrate

## Abstract

An AlGaN/GaN Schottky barrier diode (SBD) with double-heterojunction is theoretically and experimentally investigated on the GaN/AlGaN/GaN/Si-sub. The two-dimensional hole gas (2DHG) and electron gas (2DEG) are formed at the GaN-top/AlGaN and AlGaN/GaN interface, respectively. At the off-state, the 2DEH and 2DHG are partially depleted and then completely disappear. There remain the fixed positive and negative polarization charges, forming the polarization junction. Therefore, a flat electric field in the drift region and a high breakdown voltage (BV) are obtained. Moreover, the anode is recessed to reduce turn-on voltage (*V*_ON_). The low-damage ICP etching process results in the improved Schottky contacts, and a low leakgae current and a low *V*_ON_ is obtained. The fabricated SBD exhibits a BV of 1109 V with anode-to-cathode distance (*L*_AC_) of 11 μm. The fabricated SBDs achieve a low *V*_ON_ of 0.68 V with good uniformity, a high on/off current ratio ∼ 10^10^ at room temperature, a low specific on-resistance (*R*_ON,SP_) of 1.17 mΩ cm^2^, and a high Baliga’s figure-of-merit (FOM) of 1051 MW/cm^2^.

## Introduction

AlGaN/GaN heterostructure-based lateral diode is an attractive device because of the high electron mobility of the two-dimensional electron gas (2DEG), high electron saturation velocity, and high breakdown electric field [1–3]. Extensive efforts have been made to achieve a low turn-on voltage (*V*_ON_), a low reverse leakage current and a high breakdown voltage (BV) for the GaN diodes used in converters and inverters for power supplies and power factor corrections [4–12]. Various approaches have been proposed to solve the non-uniform distribution of the electric field. One of them is the field-plate (FP) technology [5, 13]. A fully recessed anode SBD with a dual field plate achieves a high breakdown voltage of 1.9 kV with 25 μm *L*_AC_ [5]_._ It can also significantly reduce turn-on voltage while maintaining high breakdown voltage. In addition, the conventional REduced SURface Field (RESURF) concept commonly employed in silicon technology has been demonstrated in GaN HEMT [14]. Moreover, the polarization junction (PJ) consisting of the two-dimensional hole gas (2DHG) above the 2DEG is proposed to improve the relationship between specific on-resistance (*R*_ON,SP_) and BV [15–18]. GaN-based devices based on the PJ concept have been demonstrated on Sapphire and SiC substrate, while the high cost and small diameters of the GaN on SiC substrates go against the mass commercial application. GaN-on-Si with a large diameter is considered as a promising choice owing to the low cost [19–22]. Therefore, the performance of the PJ diode on silicon substrates is worthy of study.

In this work, we proposed and experimentally demonstrated a GaN/AlGaN/GaN-on-Si Schottky barrier diode with double-heterojunction (DJ). The polarization-junction effect is confirmed by simulations and experiments. The flat electric field (E-field) between the anode and cathode electrodes is achieved. The ICP process to etch Schottky trench is optimized to achieve a low reverse leakage current and a low *V*_ON_ with excellent etching uniformity. The ohmic contact process is also optimized to achieve a low contact resistance (for 2DEG) based on the customized epitaxial layer (with 45 nm GaN-top). Therefore, a breakdown voltage of 1109 V is achieved for the SBDs with 11 μm *L*_AC_ and Baliga’s figure-of-merit (FOM) is 1051 MW/cm^2^. The temperature dependence and dynamic *R*_ON,SP_ performance are also investigated.

## Method and Experiment

**Fig. 1:**
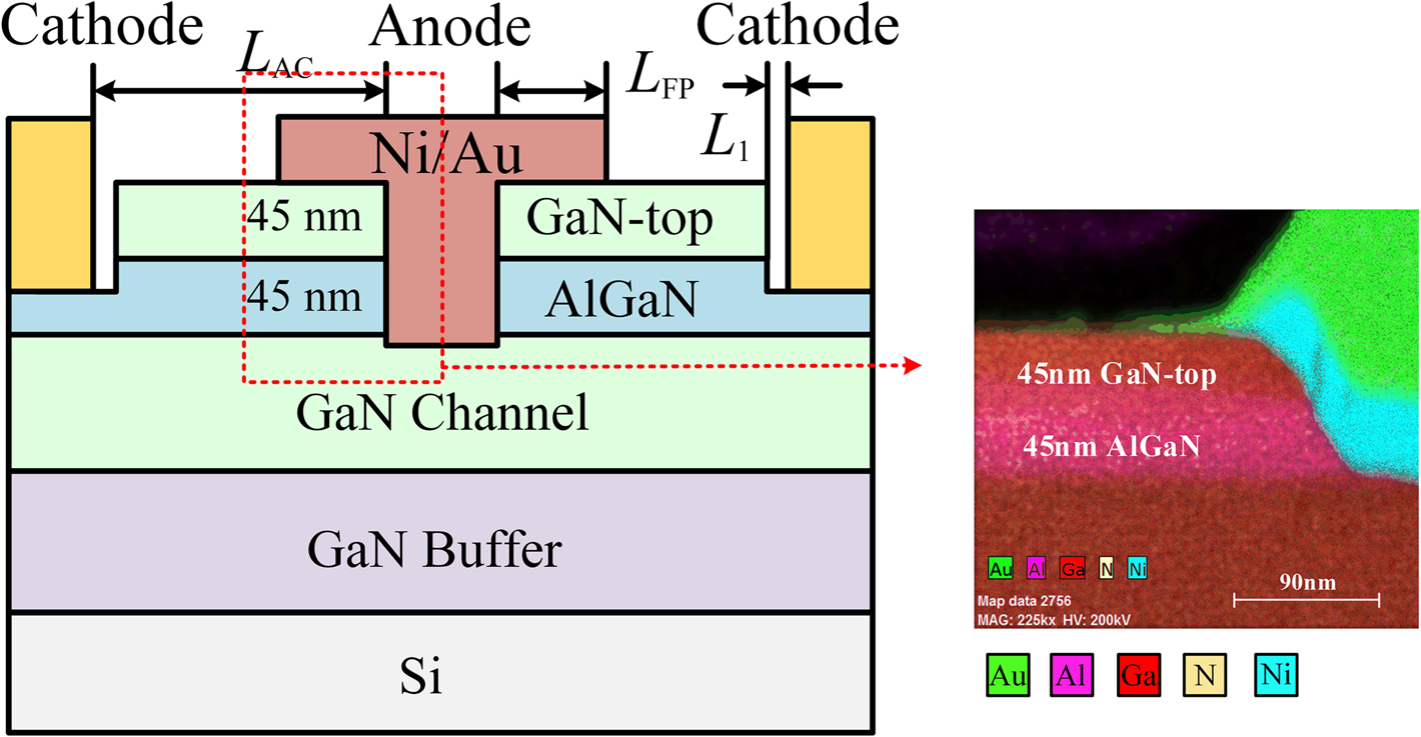
**a** Cross section of the proposed double-heterojunction AlGaN/GaN SBD and main fabrication process. *L*_AC_ is the length of anode to cathode. *L*_FP_ and *L*_1_ are 1 μm and 2 μm, respectively. **b** HR-TEM image of the anode after ICP and metal deposition

The epitaxial layer was grown by metal-organic chemical vapor deposition on 6-in p-type silicon, consisting of 3.5-μm GaN buffer layer, 150-nm GaN channel layer, 1-nm AlN interlayer, 45-nm Al_0.25_Ga_0.75_N barrier layer, and 45-nm GaN-top layer from bottom to the top. The GaN-top layer includes 35-nm p-GaN layer and 10-nm undoped GaN layer. For a given AlGaN thickness of 45 nm, the 2DHG density increases with the increase in GaN-top thickness [22]. The thick GaN-top layer is vital to the high-density 2DHG, while it goes against the low ohmic contact resistance (for 2DEG). The schematic views of the proposed double-heterojunction Schottky barrier diode (DJ SBD) are shown in Fig. 1a. The SBD fabrication started with the mesa isolation by Cl_2_/BCl_3_-based inductively coupled plasma (ICP) etching to a depth of 300 nm. Then, the ohmic trench and the Schottky anode trench were formed with the low-damage ICP etching process. The depth of the ohmic trench and the Schottky anode trench was 50 nm and 90 nm, respectively, which was confirmed by using atomic force microscopy (AFM). Tetramethylammonium hydroxide (TMAH) solution at 85 °C for 15 min was introduced to remove the post-etching residues and to modify the trench sidewall after finishing the etching process [23]. Then, the annealing at 400 °C for 10 min in N_2_ ambient was carried out. The ohmic cathode was subsequently formed by e-beam evaporated Ti/Al/Ni/Au (20/140/55/45 nm), annealed at 870 °C for 35 s in N_2_ ambient, with a contact resistance (*R*_C_) of 0.49 Ω·mm. Finally, anode metal and the interconnections were deposited by Ni/Au to complete the fabrication flow. The devices featured various *L*_AC_ from 7 to 11 μm. Figure 1b shows the high-resolution cross-section TEM image of the anode after ICP and metal deposition, and the layer structure was observed clearly.

**Fig. 2:**
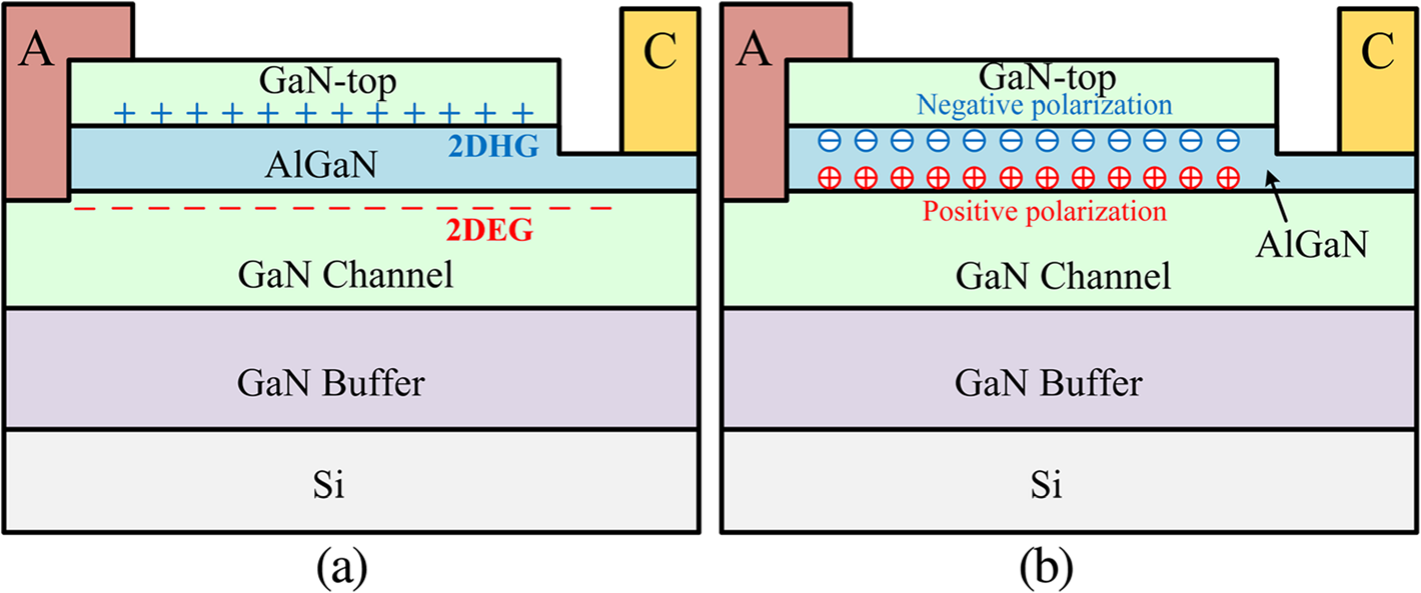
Operation mechanism analysis of DJ SBDs **a** zero-bias and **b** reverse-bias

**Fig. 3:**
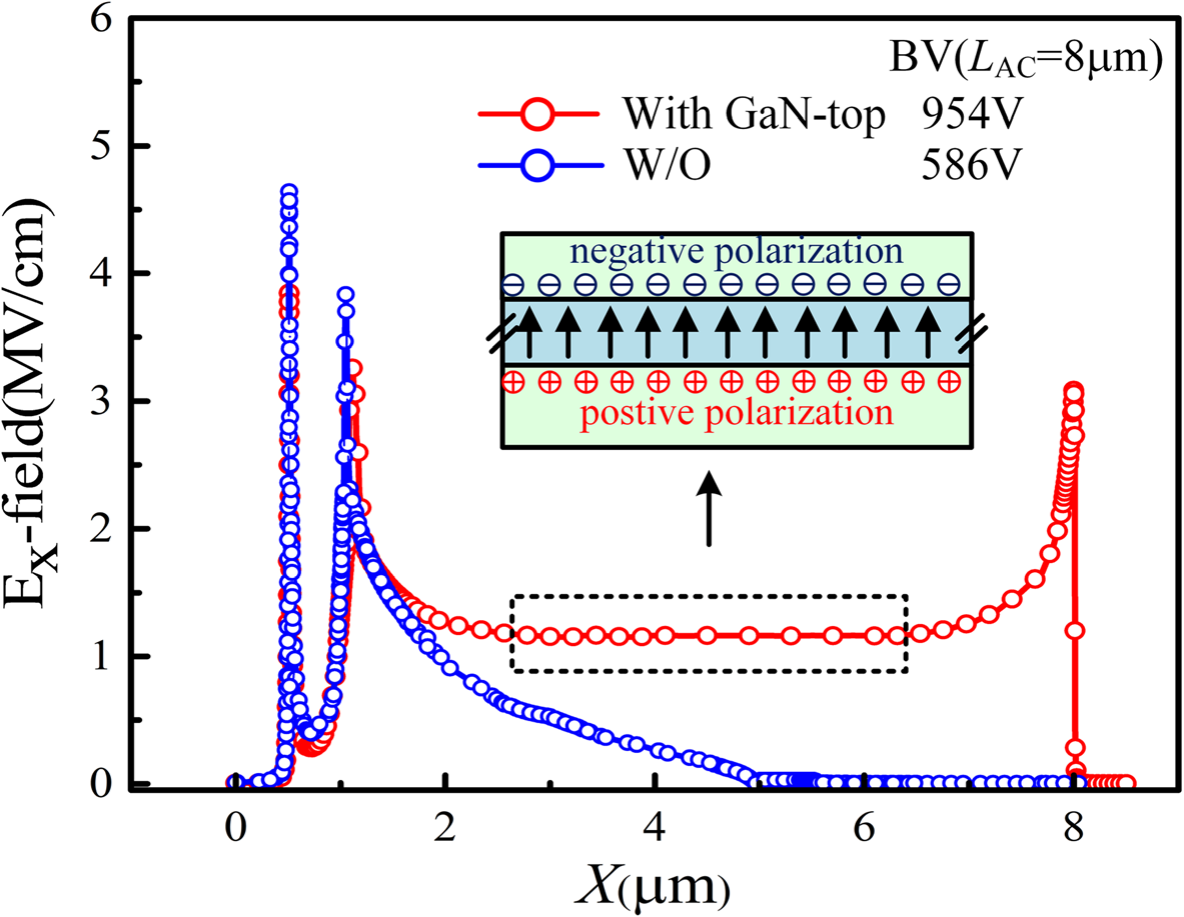
Electric field distribution along the AlGaN/GaN channel heterointerface by TCAD simulation. The Al fraction is defined as 0.25. The net acceptor (deep level) density in the buffer layer is set to be 1.5 × 10^16^ cm^−3^ and the energy level is 0.45 eV below the conduction band minimum. The acceptor density of the AlGaN/GaN interface is set to be 6 × 10^12^ cm^−3^ and the energy level is 0.23 eV below the conduction band minimum

The 2DEG is induced by the positive polarization charges along the AlGaN/GaN interface. The upper GaN/AlGaN interface has negative polarization charges and hence generates 2DHG at the upper interface [15]. The gap between the drift region and the cathode (*L*_1_) is used to cut down the hole current path as shown in Fig. 2. We have only investigated the influence of *L*_1_ from 2 to 3 μm on the forward and reverse blocking characteristics due to the limit of the original layout design. The *V*_ON_ and *R*_ON,SP_ show no change because *L*_1_ does not affect the Schottky contact and electron current path. In addition, the BV decreases slightly with the increase in *L*_1_ because of the shortened drift region. The operation mechanism of the DJ SBDs under forward bias is almost the same as the conventional SBDs, meaning that 2DHG hardly affects the electron current path from the cathode to the anode. With the increasing reverse bias voltage, the 2DEG and 2DHG are fully depleted. There remain fixed positive and negative polarization charges, which forms the polarization junction. As a result, a flat E-field distribution between the cathode and anode is obtained (Fig. 3).

As shown in the Fig. 3, the breakdown characteristic and polarization-junction mechanism were confirmed by 2-D Sentaurus TCAD from Synopsys. We had accounted for several important physical phenomena in simulation, including bandgap narrowing, polarization, electron/hole mobility, impact ionization and SRH recombination.

Hall effect measurement was adopted to determine the 2DHG or 2DEG density and mobility values [22]. The measurements were performed by Van der Pauw method at room temperature. To measure 2DHG according to Ref. [17], Hall measurement samples were fabricated with Ni/Au ohmic contacts. The density and mobility of the 2DHG were 9 × 10^12^ cm^−2^ and 15 cm^2^/V s, respectively. The 2DEG was measured by the samples with recessing GaN-top and partially AlGaN fabricated with Ti/Al/Ni/Au ohmic contacts (for 2DEG). The density and mobility of the 2DEG were 8.5 × 10^12^ cm^−2^ and 970 cm^2^/V s, respectively. The Hall measurements showed that the hole mobility was still much lower than the bulk mobility over 100 cm^2^/V s. The degradation of mobility was attributed to the diffusion of Mg from the p-GaN to the undoped GaN during the MOCVD growth.

## Results and Discussion

**Fig. 4:**
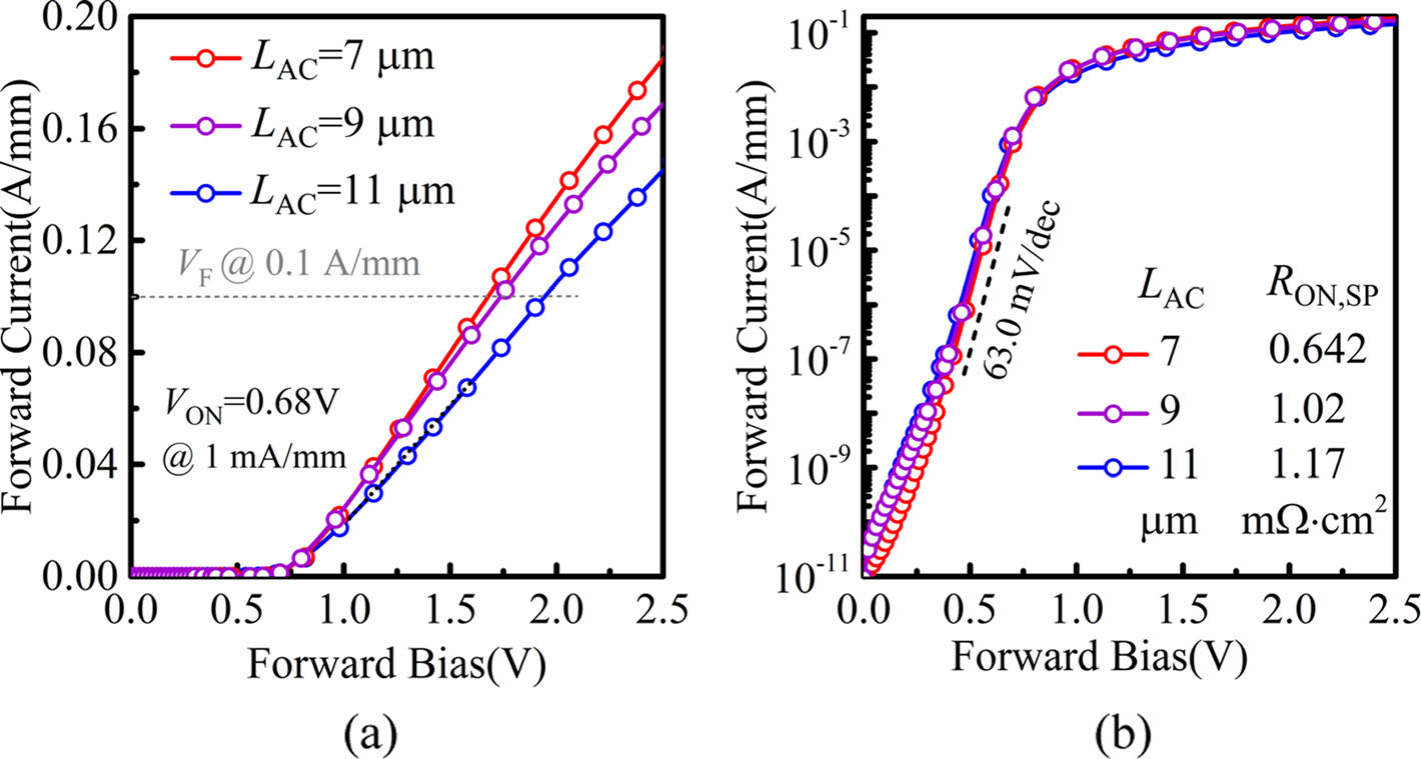
Measured forward bias *I-V* characteristics of DJ SBDs in **a** linear and **b** semi-log scale with different *L*_AC_

The measured *I-V* forward characteristics of the SBDs with various *L*_AC_ are plotted in Fig. 4a and b. The turn-on voltage (*V*_ON_) is 0.68 V with a small variation of 0.02 V. The ideality factor and the barrier height of the SBDs are calculated as 1.44 ± 0.15 and 0.76 ± 0.04 eV, respectively. Figure 4a shows that the high forward current density of 183 mA/mm and 144 mA/mm (@ forward bias of 2.5 V) and the on-resistance of 0.642 and 1.17 mΩ cm^2^ are achieved at *L*_AC_ = 7 and 11 μm, respectively. In addition, an excellent on/off current ratio ∼ 10^10^ is obtained as shown in Fig. 4b. The subthreshold slope (SS) is 63.0 mV/dec, which is close to the ideal SS (59.6 mV/dec).

**Fig. 5:**
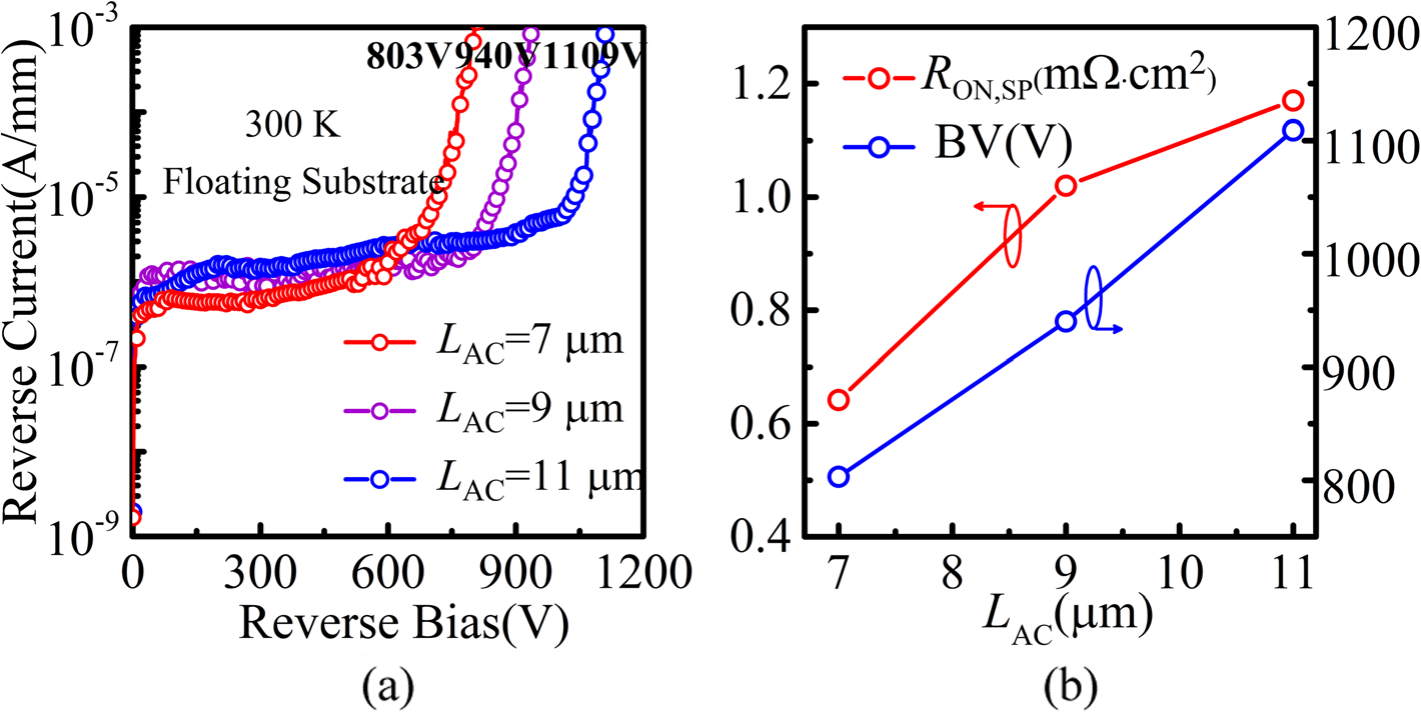
**a** Measured reverse blocking *I-V* characteristics of DJ SBDs with different *L*_AC_ (**b**) *R*_ON,SP_ and BV as the functions of *L*_AC_

Figure 5a shows the measured reverse blocking *I-V* characteristics of the SBDs with various *L*_AC_ at 300 K. The breakdown voltage of the devices with different *L*_AC_ is 803 V, 940 V, and 1109 V, respectively, at a leakage current of 1 mA/mm. The densities of 2DEG and 2DHG are supposed the same during the simulation. However, the experimental results show that the densities of 2DHG (9 × 10^12^ cm^−2^) are slightly higher than those of 2DEG (8.5 × 10^12^ cm^−2^). The difference between the fixed positive and negative polarization charges during the off-state affects the charge balance and thus degrades the breakdown voltage. The influence of the *L*_AC_ on the BV and the *R*_ON,SP_ is shown in Fig. 5b. A near linear relationship between BV and *L*_AC_ is obtained, implying the relative flat lateral E-field in the drift region. Owing to the polarization-junction effect, the device demonstrates a high Baliga’s figure-of-merit (FOM = BV^2^/*R*_ON,SP_) of 1051 MW/cm^2^ (@ *L*_AC_ = 11 μm).

**Fig. 6:**
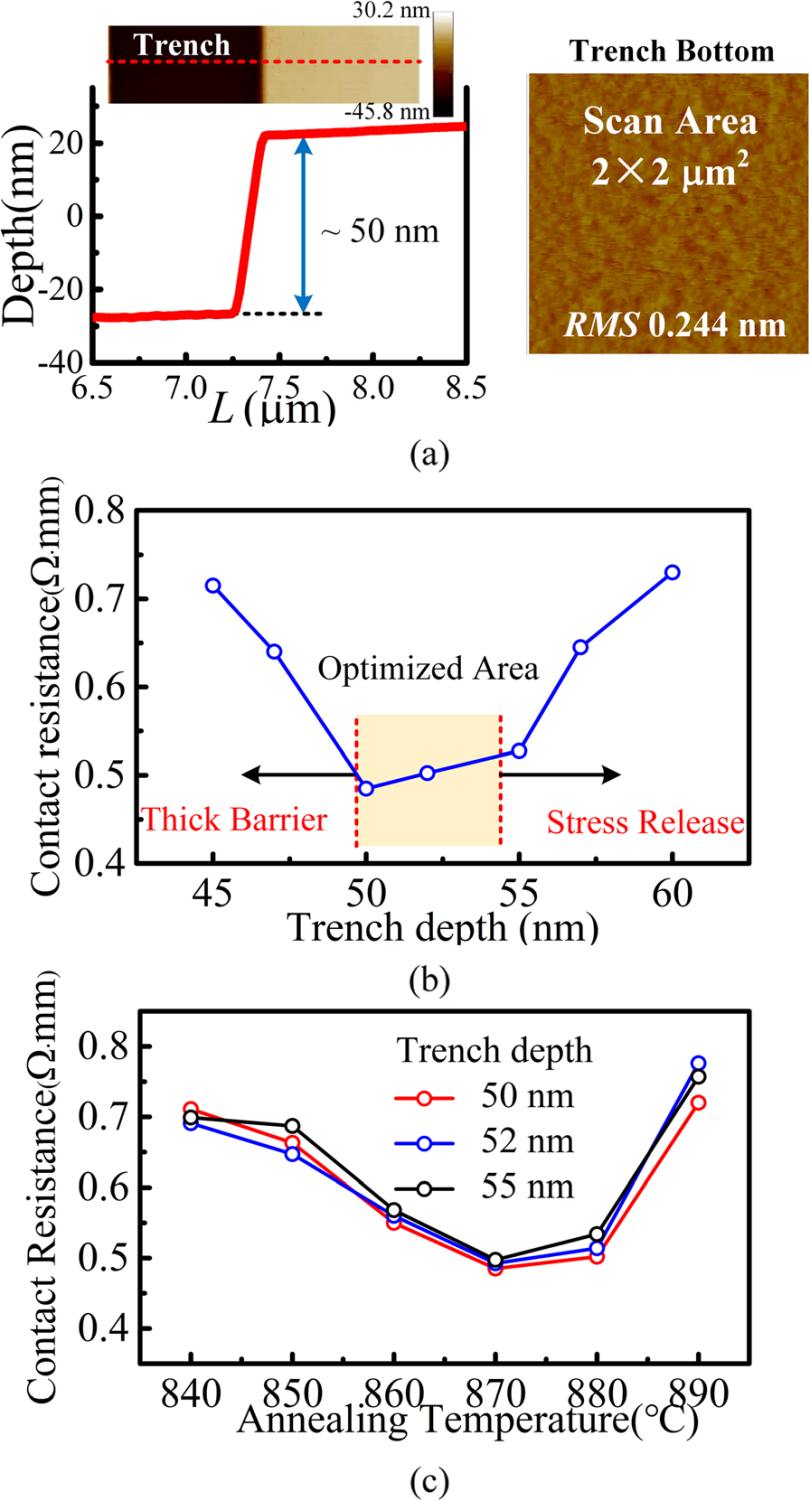
**a** AFM morphology of the trench after ICP etching. **b** Influence of etch depth on ohmic contact resistance by TLM test. **c** Contact resistance as a function of annealing temperature with the trench depth from 50 to 55 nm. The annealing time was 35 s

The etching process is vital to the high-quality Schottky interface and ohmic contact. Figure 6a shows the surface morphology of the recessed trench after the ICP etching (@ 5 °C) and the TMAH solution. The etch rate is approximately 4.9 nm/min, and the final selected recipe is with a Cl_2_ of 4 sccm, an ICP power of 50 W, and an RF power of 15 W. The root mean square (RMS) roughness is 0.244 nm with the scan area of 2 × 2 μm^2^.

Because the customized epitaxial layer includes 45 nm GaN-top layer and 45 nm AlGaN layer, the ohmic contact (for 2DEG) process is different from the conventional SBDs. Without recessing both GaN-top and AlGaN barrier layers, low contact resistance is difficult to be achieved by annealing because of the potential barrier between the ohmic metal and the 2DEG. However, if the barrier is over recessed, the stress release leads to a reduction in the 2DEG concentration. The extra processes are adopted to reduce the ohmic contact resistance. The surfaces of the samples are treated by the HCl solution to remove native oxide layer before deposition. In addition, the plasma surface treatment is adopted (ICP power 50 W BCl_3_ 10 sccm 3 min) to introduce surface donor states [24]. Figure 6b demonstrates the dependence of the contact resistance on the trench depth. The optimized depth is obtained from 50 to 55 nm. The high temperature rapid thermal annealing (RTA) for the Ti/Al/Ni/Au contact is investigated in Fig, 6c. The annealing temperature is from 840 to 890 °C, and the 870 °C results in the lowest contact resistance. Annealing at high temperature, i.e., 870 °C, is conducive to the formation of TiN at the Ti/nitride interface. However, higher temperature (e.g., 890 °C) increases the interdiffusion of Au and Al, which is disadvantageous for the formation of good ohmic contacts.

**Fig. 7:**
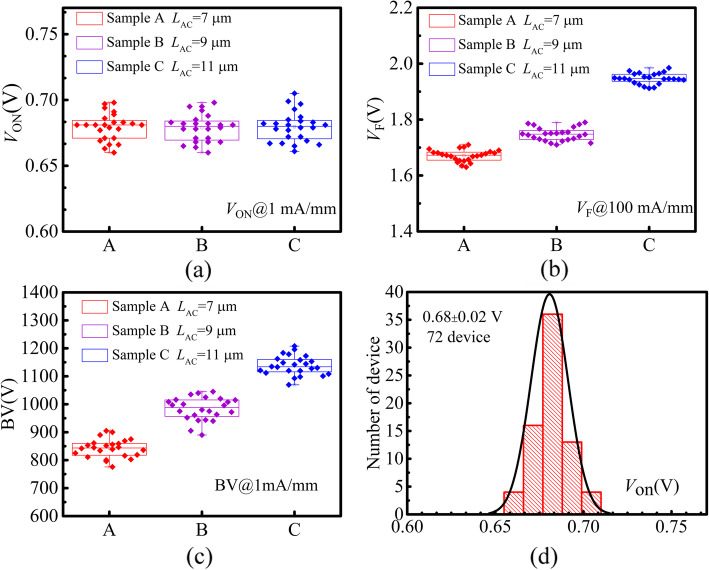
Statistical plots of **a** turn-on voltage, b forward voltage, and **c** breakdown voltage extracted from 72 SBDs with *L*_AC_ of 7, 9, and 11 μm fabricated in 3 separate process runs. **d** Distribution of *V*_ON_ for 72 devices

Figure 7a–c exhibit the statistical plots of the static characteristics including *V*_ON_, *V*_F_, and BV. The data are extracted from 72 SBDs with *L*_AC_ of 7, 9, and 11 μm fabricated in 3 separate process runs. The devices display stable forward turn-on characteristics and the *V*_ON_ is independent with *L*_AC_, because *V*_ON_ is mainly determined by the Schottky contact. The low-damage ICP etching process, the precisely controlled trench depth, and the high-quality Schottky interface contribute to the excellent uniformity of the *V*_ON_ and *V*_F_. In addition, with the increase in *L*_AC_ (from 7 to 11 μm), there is a monotonical increase (∼ 100 V/μm) in the BV observed in proposed structures. Figure 7d shows the histogram statistics of the *V*_ON_ for 72 devices, and the mean value is 0.68 V with a small standard derivation of 0.02 V.

**Fig. 8:**
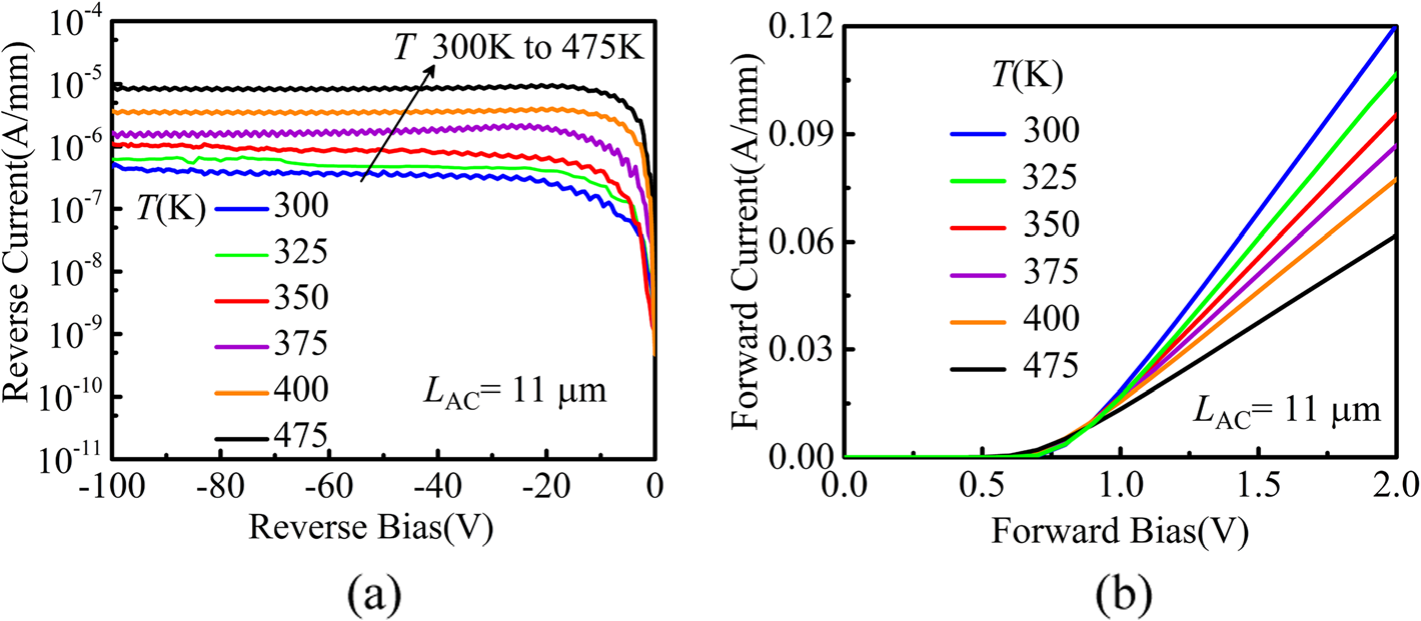
**a** Reverse leakage current and **b** forward characteristics for the DJ SBDs at different temperatures

The temperature dependence of the reverse and forward characteristics is assessed in Fig. 8. As shown in Fig. 8a, an increase in ambient temperature from 300 to 475 K results in an increase in the *R*_ON,SP_ by a factor of 1.94.

**Fig. 9:**
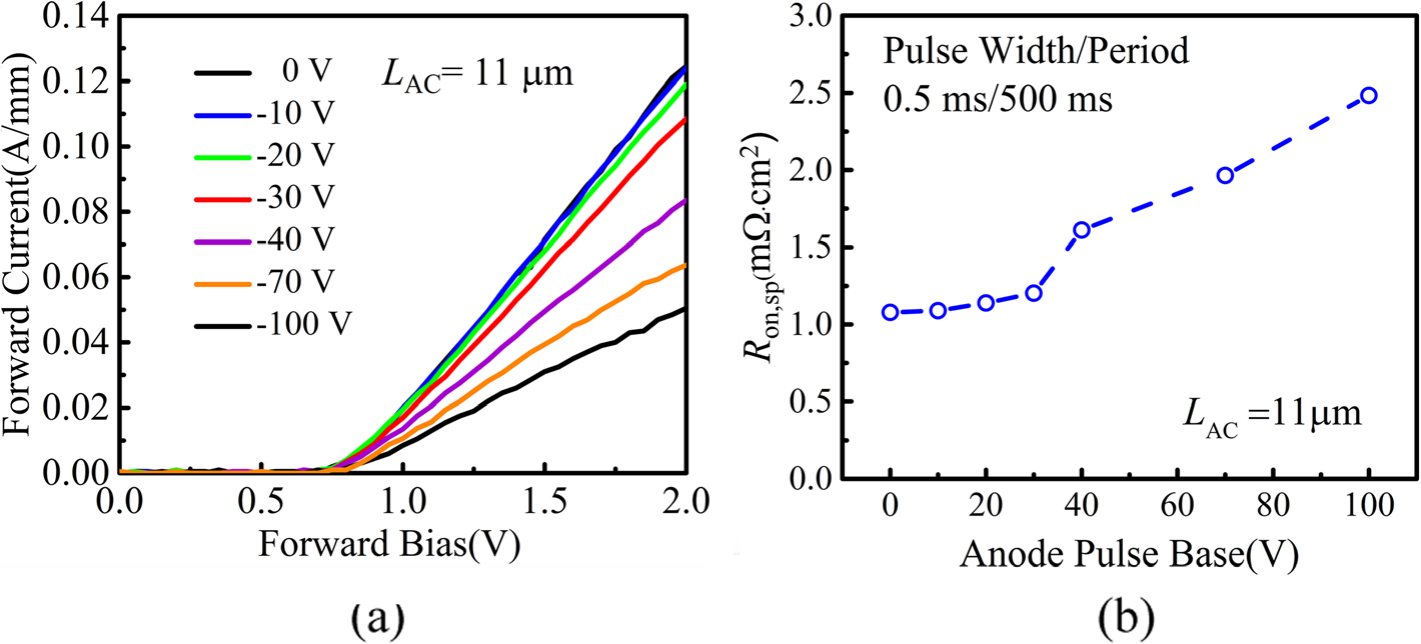
**a***I-V* characteristics under pulse measurement. **b** Extracted *R*_ON,SP_ versus anode pulse base with pulse width/period = 0.5 ms/500 ms

The dynamic characteristics of the DJ SBDs are measured by Agilent B1505A power device analyzer. The anode pulse quiescent bias points are set to be − 10 V, − 20 V, − 30 V, − 40 V, − 70 V, and − 100 V, with the anode pulse width and period of 0.5 ms/500 ms. Figure 9b shows the dynamic *R*_ON,SP_ as a function of the stress voltage. The dynamic *R*_ON,SP_ even at 100 V reserve stress voltage is just 1.18 times of the one without reverse stress, which is comparable to Ref. [8]. The limited increase in the dynamic *R*_ON,SP_ contributes to the reduction in interface state. The degradation of dynamic *R*_ON,SP_ needs further work.

**Fig. 10:**
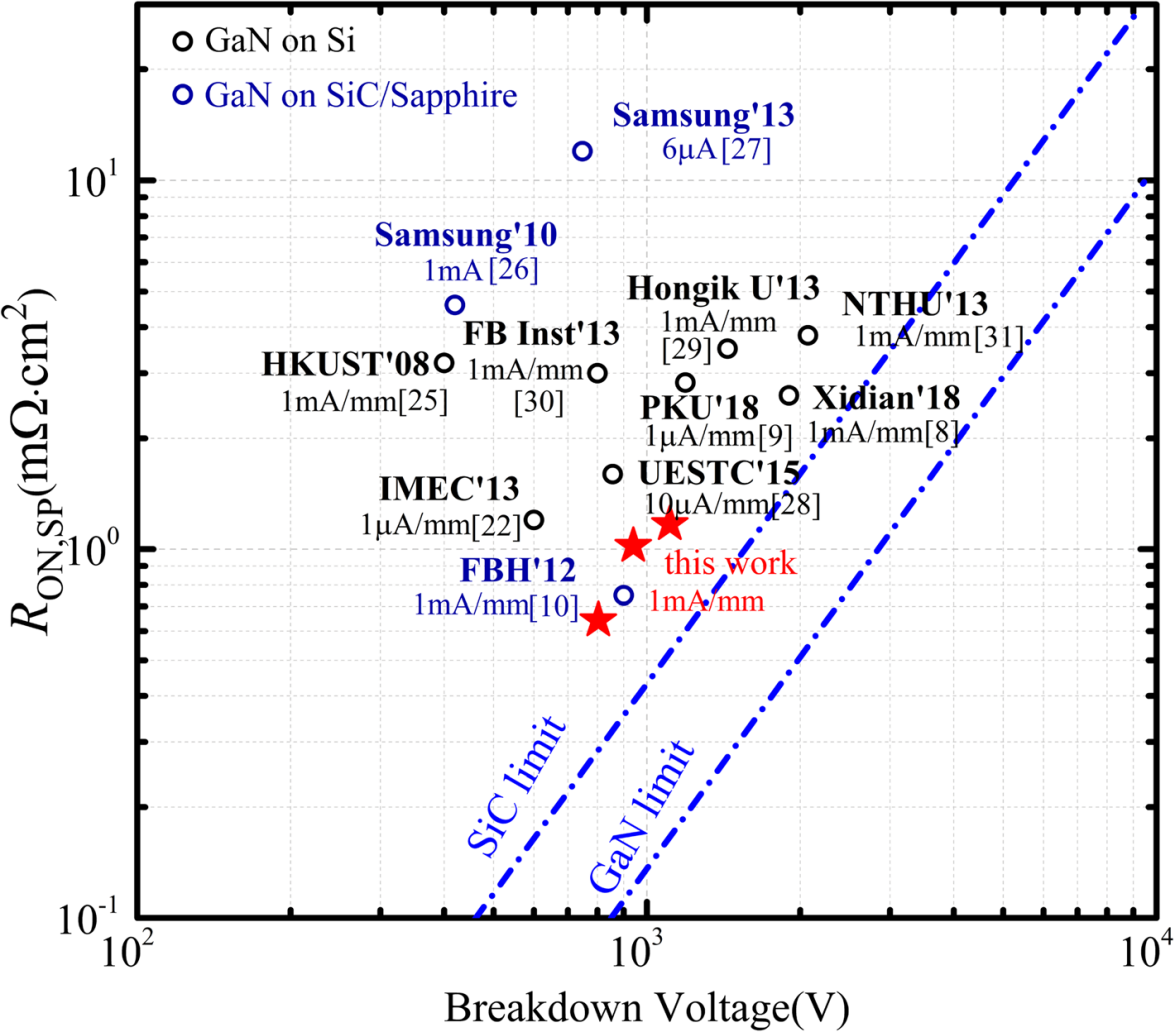
Benchmark plot of BV versus *R*_ON,SP_ of GaN power diode on SiC/sapphire/Si substrates. The reverse leakage used to define the breakdown is also given

Figure 10 presents the benchmark plot of BV versus *R*_ON,SP_ for GaN power diode on Si/SiC/sapphire substrates [8, 10, 22, 25–31]. The proposed device with *L*_AC_ of 11 μm demonstrates a BV of 1109 V with a corresponding *R*_ON,SP_ of 1.17 mΩ cm^2^, leading to a high Baliga’s FOM of 1051 MW/cm^2^. This value is the best results among the lateral GaN power diode on Si substrate.

## Conclusion

A double-heterojunction GaN/AlGaN/GaN-on-Si SBD with a high figure of merit is fabricated. The low-damage ICP etching process results in the optimized ohmic and Schottky contacts for the proposed device. Therefore, a low *V*_ON_ of 0.68 V with good uniformity and a low *R*_ON,SP_ of 1.17 mΩ cm^2^ are obtained in the device with *L*_AC_ of 11 μm. A high Baliga’s FOM of 1051 MW/cm^2^ is achieved due to the polarization-junction effect. The high performance together with the low-cost GaN-on-Si technology exhibits great potential for future power applications.

## Data Availability

All data generated or analyzed during this study are included in this article.

## Abbreviations

SBD: Schottky barrier diode
2DEG/2DHG: Two-dimensional electron/hole gas
MOCVD: Metal-organic chemical vapor deposition
ICP: Inductively coupled plasma
TEM: Transmission electron microscope
AFM: Atomic force microscope
BV: Breakdown voltage
*R*_ON,SP_: Specific on-resistance
*V*_ON_: Turn-on voltage
FOM: Figure-of-merit
